# Targeting HER3 to overcome EGFR TKI resistance in NSCLC

**DOI:** 10.3389/fimmu.2023.1332057

**Published:** 2024-01-04

**Authors:** Qiuqiang Chen, Gang Jia, Xilin Zhang, Wenxue Ma

**Affiliations:** ^1^ Key Laboratory for Translational Medicine, The First Affiliated Hospital, Huzhou University, Huzhou, Zhejiang, China; ^2^ Department of Medical Oncology, Henan Provincial People’s Hospital, People’s Hospital of Zhengzhou University, Zhengzhou, Henan, China; ^3^ Department of Medicine, Moores Cancer Center, and Sanford Stem Cell Institute, University of California, San Diego, La Jolla, CA, United States

**Keywords:** non-small cell lung cancer (NSCLC), epidermal growth factor receptor (EGFR), tyrosine kinase inhibitors (TKIs), receptor tyrosine kinases (RTKs), resistance, human EGFR3 (HER3), antibody-drug conjugates (ADCs), Patritumab Deruxtecan (HER3-DXd)

## Abstract

Receptor tyrosine kinases (RTKs) play a crucial role in cellular signaling and oncogenic progression. Epidermal growth factor receptor tyrosine kinase inhibitors (EGFR TKIs) have become the standard treatment for advanced non-small cell lung cancer (NSCLC) patients with EGFR-sensitizing mutations, but resistance frequently emerges between 10 to 14 months. A significant factor in this resistance is the role of human EGFR 3 (HER3), an EGFR family member. Despite its significance, effective targeting of HER3 is still developing. This review aims to bridge this gap by deeply examining HER3’s pivotal contribution to EGFR TKI resistance and spotlighting emerging HER3-centered therapeutic avenues, including monoclonal antibodies (mAbs), TKIs, and antibody-drug conjugates (ADCs). Preliminary results indicate combining HER3-specific treatments with EGFR TKIs enhances antitumor effects, leading to an increased objective response rate (ORR) and prolonged overall survival (OS) in resistant cases. Embracing HER3-targeting therapies represents a transformative approach against EGFR TKI resistance and emphasizes the importance of further research to optimize patient stratification and understand resistance mechanisms.

## 1 Introduction

Lung cancer, particularly non-small cell lung cancer (NSCLC), remains a pivotal global health challenge. In 2023, it is projected that the United States will face an estimated 238,340 new NSCLC cases, resulting in a staggering 127,070 deaths, as reported by the Cancer.Net Editorial Board (www.cancer.net). NSCLC comprises approximately 85% of all lung cancer cases and is primarily categorized into adenocarcinoma, squamous cell carcinoma, or large cell carcinoma (www.cancer.org).

The hallmark of NSCLC is uncontrolled cellular proliferation within lung tissues, resulting in tumor formation (1–3). Surgical intervention remains the gold standard for patients diagnosed at early stages (stages I to II), however, the specter of recurrence persists, with rates fluctuating between 30% to 55% within five years post-surgery (4). Pignon et al. emphasized the enduring challenges of recurrence and mortality across NSCLC stages (5).

Despite advancements in therapy, the overall survival (OS) metrics for NSCLC have remained disappointingly stagnant. A study by Goldstraw et al. in 2016 portrayed a bleak picture, illustrating decreasing survival rates with disease progression (6). This underscores the urgent need for innovative therapeutic strategies.

Emerging molecular insights have identified the epidermal growth factor receptor (EGFR) gene as a pivotal player in the pathological development of NSCLC (7, 8). Aberrations in EGFR can trigger abnormal cellular growth (9–12). Distinct mutations, associated with varying NSCLC progression, are more prevalent in certain populations (13, 14). Key studies have revealed that a significant proportion of NSCLC patients harbor EGFR mutations (15, 16), with the common types including EGFR 19 deletions (17, 18), EGFR exon 21 L858R point mutations EGFR exon 20 insertions, T790M-like mutations and P-loop αC-helix compression (PACC) mutations (17–19). A multicenter study by the National Network Genomic Medicine in Germany grouped these mutations into three major categories: uncommon mutations (G719X, S7681, L861Q, and combinations), exon 20 insertions, and very rare EGFR mutations (very rare single point mutations, compound mutations, exon 18 deletions, exon 19 insertions) (18).

The prevalence of EGFR mutations in NSCLC patients varies by region, with an overall rate of 17.2%. In Southeast Asia, it stands within the range of 40% to 60%, while North Africa records a lower prevalence at 18%. In Caucasian populations, the prevalence falls between 10% and 20%, and in the Middle East, it is slightly higher than that observed in Caucasian populations (20, 21). These regional disparities underscore the importance of considering genetic factors in NSCLC diagnosis and treatment strategies.

Patients whose tumors exhibit EGFR mutations initially respond favorably to first-generation EGFR tyrosine kinase inhibitors (TKIs) such as erlotinib and gefitinib (22, 23). However, the development resistance, particularly through secondary EGFR mutations like T790M, hampers long-term therapeutic success (24–26). Osimertinib, a pioneering third-generation EGFR-TKI, emerged as a response to the T790M mutation in patients who developed resistance to the earlier TKIs, providing a lifeline for those battling advanced NSCLC (25, 27). Nonetheless, even these promising treatments are not entirely immune to resistance, underscoring the need for alternative therapeutic modalities (28, 29).

An intriguing observation has been the elevated HER3 signaling in NSCLC patients who develop resistance to EGFR TKIs, suggesting HER3’s ancillary role in the resistance modulation (30, 31). This has shifted the research spotlight onto HER3, not just as an EGFR family member, but as a potential linchpin in the resistance mechanism.

Currently, the HER3 receptor, an EGFR family member, is garnering significant attention. As research delves deeper, the role of HER3 in EGFR TKI resistance becomes increasingly apparent, presenting a tantalizing therapeutic target (32, 33). This review delves into the promising realm of targeting HER3 against EGFR TKI resistance in NSCLC. We aim to demystify the intricate EGFR-HER3 relationship, evaluate HER3-focused therapeutic strategies, and champion the potential of HER3 inhibition as a novel approach against EGFR-driven NSCLC.

## 2 The significance of HER3 in the EGFR family framework

The HER family, a subset of the erythroblastic oncogene B (ERBB) category, plays a pivotal role in cellular regulation, comprising receptors such as EGFR (ERBB1/HER1), HER2 (ERBB2), HER3 (ERBB3), and HER4 (ERBB4), orchestrate critical cellular processes, including growth, survival, and differentiation (34, 35). Within this family, HER3 has increasingly been recognized for its distinct role in the pathogenesis of NSCLC and its implications for patient prognosis.

Studies have consistently shown that high HER3 expression is associated with advanced NSCLC and poorer outcomes, suggesting its value as an independent prognostic indicator (36, 37). The overexpression of HER3’s ligand, heregulin (HRG), further contributes to the disease’s aggressiveness by enhancing tumor proliferation and metastatic capacity (38). HRG-mediated activation of HER3 leads to downstream PI3K/AKT pathway signaling, promoting cell survival, and imparting resistance to EGFR-targeted therapies, a cornerstone of the NSCLC treatment (39, 40).

The clinical impact of HER3 is also exacerbated by its co-expression with other EGFR family members, notably HER2 and EGFR, which is linked to a more aggressive NSCLC phenotype and therapeutic resistance (39, 41, 42). Current research into the molecular mechanisms of HER3 has revealed its role in oncogenic signaling cascades that underpin NSCLC progression, thereby highlighting the potential of HER3 as a therapeutic target (42).

**Figure 1:**
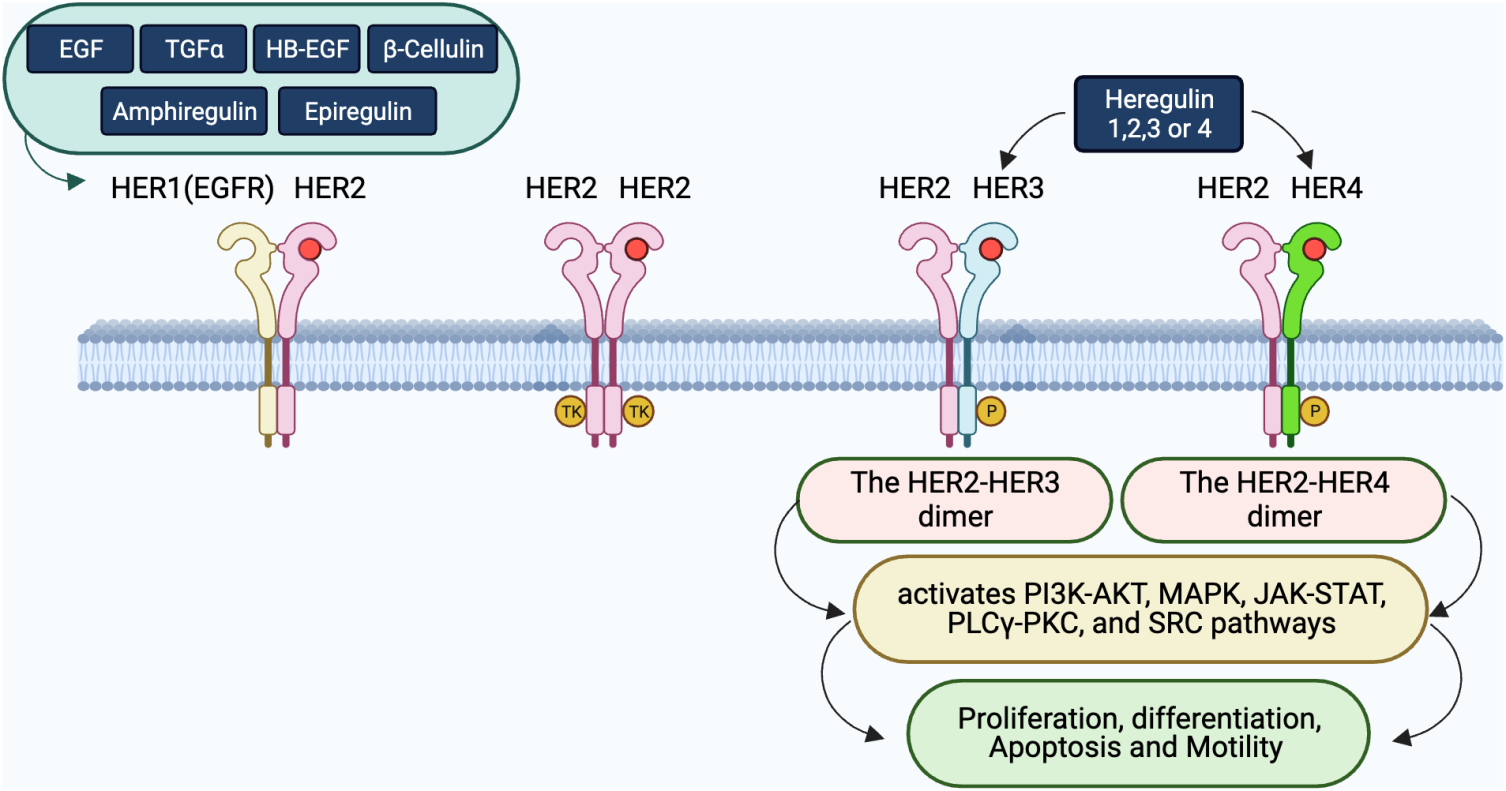
Interactions and dimerization within the HER receptor family. The diagram displays the complex ligand-receptor engagements within the HER family. Ligands including EGF, TGFα, HB-EGF, β-Cellulin, Amphiregulin, Epiregulin, and heregulin (HRG) bind to the HER receptors, inducing diverse dimeric interactions. Specifically, HER1 associates with HER2, while HER2 can either create homodimers or bind to HER3 or HER4 upon HRG’s presence. The significance of these dimers, particularly HER2-HER3 and HER2-HER4, in triggering crucial downstream signaling pathways such as PI3K-AKT, MAPK, JAK-STAT, PLCγ-PKC, and SRC, that regulate cellular processes like proliferation, differentiation, apoptosis, and movement, is emphasized.

Despite its low intrinsic kinase activity, HER3’s capacity to dimerize, particularly with HER2, is essential for signaling, which is critical for cell growth and survival (40, 43–45). This dimerization often intensified during EGFR TKI treatment, leads to therapy resistance, emphasizing the complexity of targeting the EGFR/HER3 axis (45, 46). The interplay between abnormal EGFR mutations and increased HER3 activity underlines the necessity for multi-receptor targeting strategies to overcome the resistance (47). These intricate relationships, especially the dynamic with HER2 and the consequential signaling pathways they initiate, are further elaborated, and visualized in **Figure 1**.

**Figure 2:**
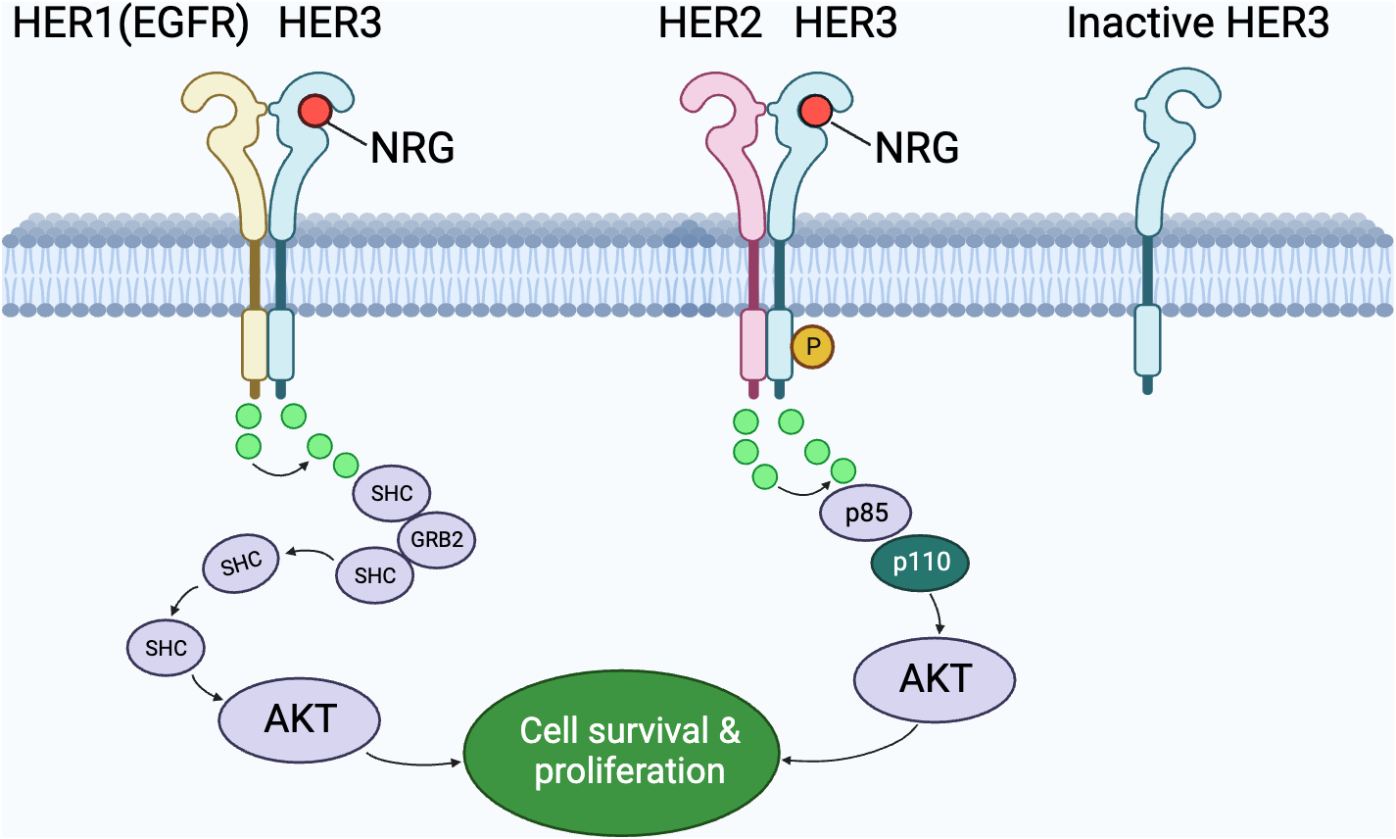
The interplay of HER family members in cell signaling pathways. This graphic depicts the dimerization interactions between HER1 (EGFR) and HER3, as well as between HER2 and HER3. The emphasis is on HER3’s significance in both its operational and dormant states. An operational HER3 forms dimers with fellow members to convey signals through intermediaries like SHC, p85, and p110, whereas a dormant HER3 remains detached. Notably, the interaction of HER2 and HER3, when the ligand NRG is present, results in the AKT pathway’s activation, promoting cell survival and proliferation. The role of various molecular intermediaries in transmitting these signals is also portrayed.

After understanding the fundamental ligand-receptor interactions within the HER family as illustrated in **Figure 1**, it’s essential to delve deeper into the specific dimerization dynamics that play a pivotal role in cellular signaling. **Figure 2** offers a more focused look at these interactions, emphasizing the key roles of HER3 in both its active and dormant states.

## 3 EGFR TKI resistance mechanisms

EGFR TKIs have significantly changed the treatment landscape of NSCLC for patients with EGFR mutations by targeting the critical tumorigenic EGFR pathway. However, the clinical efficacy of these agents is often compromised by the emergence of resistance mechanisms, presenting an ongoing and significant obstacle in NSCLC management (48–50). The most well-characterized of these resistance mechanisms is the acquisition of secondary mutations in the EGFR gene, with the T790M mutation being exemplary. This mutation changes the EGFR protein structure, thereby increasing its affinity for ATP and diminishing the effectiveness of first- and second-generation EGFR TKIs. This alteration facilitates continued tumor cell proliferation and survival by effectively bypassing the TKI’s inhibitory effects (29, 47, 51–55).

However, tumor cell adaptability extends beyond genetic mutations. NSCLC cells can invoke bypass signaling pathways or amplify other receptor tyrosine kinases, such as HER2, to sustain proliferative signaling. The overexpression of HER2 leads to ligand-independent activation of downstream pathways, particularly the PI3K/AKT and MAPK pathways, contributing to ongoing cell growth and survival, thereby mitigating the effects of EGFR inhibition (50, 56–58). These resistance mechanisms are diverse and multifaceted, as summarized in **Table 1**.

**Table 1:** Summary of EGFR TKI Resistance Mechanisms in NSCLC.

Mechanism	Description	Impact on Resistance	References
Secondary EGFR Gene Mutations	T790M “gatekeeper” mutation alters EGFR protein structure, enhancing its ATP affinity	Reduces efficacy of EGFR TKIs, promoting tumor proliferation	(55, 59, 60)
Bypass Signaling	Activation of alternative pathways (e.g., MET) and amplification of other receptors (e.g., HER2)	Bypasses the effects of EGFR TKIs, ensuring continued growth and survival of cancer cells	(12, 57, 61, 62)
Epithelial-Mesenchymal Transition (EMT)	Transition imbues cells with stem-like properties, increased mobility, and enhanced resistance	Increases resilience against TKIs and promotes tumor invasiveness	(63–65)
HER3 Activation	Upregulation and interaction with PI3K/AKT and HER2 pathways	Strengthens cell signaling, fostering cellular adaptability and resistance	(39, 66, 67)
Growth Factors Activation	Growth factors (EGF, VEGF, PDGF, FGF) binding and activating tyrosine kinase enzymes	Promotes cellular division and growth, counteracting TKI effects	(68–70)

The complexity of resistance is further exemplified by the role of HER3. Unlike its more active family members, HER3 lacks intrinsic kinase activity but becomes a potent signaling entity upon heterodimerization with other receptors such as HER2, EGFR, and MET. These heterodimers act as intricate molecular switches that can engage various intracellular signaling cascades, contributing to a robust and resilient network of proliferative signals that enhance the cell’s ability to withstand targeted therapy (39, 41). Recent research on ALK+ non-small cell lung cancer sheds light on HER3’s significant role in resistance. In contrast to other active receptors, HER3 becomes a powerful signaling agent when paired with receptors like HER2, EGFR, and MET, leading to resilient growth signals that resist targeted therapies. A study on ALK inhibitors in ALK+ NSCLC unveiled HER3’s involvement in resistance. Co-targeting ALK and HER3 with inhibitors hindered colony growth and reduced pAKT levels, highlighting the potential of joint ALK and HER3 targeting as a promising avenue in overcoming resistance (71).

Clinical investigations have started to illuminate the significance of HER3 heterodimers as biomarkers for resistance. Notably, the co-existence of HER3 with HER2 or MET has been associated with poor therapeutic outcomes, indicating their potential utility as predictive markers for drug resistance. Furthermore, they present themselves as novel targets for the next generation of targeted therapies (31, 38, 71). For instance, recent clinical observations have highlighted the correlation between increased levels of HER3/HER2 heterodimers and a diminished response to EGFR TKIs. Such correlations are opening new avenues for the development of therapeutic strategies aimed at disrupting these heterodimers (33, 72, 73).

The table presents a concise summary of the multifaceted resistance mechanisms that have been characterized in NSCLC, emphasizing the diverse strategies employed by cancer cells to evade the effects of TKIs.

Addressing the multifaceted nature of EGFR TKI resistance requires an integrated approach that combines molecular profiling with an in-depth understanding of the cellular signaling landscape. This approach is vital for the development of personalized treatments that can adapt to the evolving genetic context of each patient’s cancer (74–76). The recognition of HER3 heterodimerization’s role in resistance mechanisms is a testament to the continued need for innovation in the field of targeted therapy.

In conclusion, EGFR TKIs have marked a paradigm shift in NSCLC treatment, yet the battle against resistance is ongoing. The elucidation of complex resistance mechanisms, especially involving HER3 and its heterodimers, is critical for the development of novel therapeutic strategies. These strategies will not only need to counteract existing resistance pathways but also preemptively address potential future mechanisms of resistance to improve long-term patient outcomes.

## 4 Strategies and methods of targeting HER3

The introduction of EGFR TKIs has marked a significant milestone in NSCLC treatment for patients with EGFR mutations. However, resistance to these agents, notably from secondary mutations such as T790M, has necessitated novel therapeutic strategies. Within this context, the HER3 receptor has become a focal point due to its involvement in resistance to EGFR TKIs (38, 75–77).

HER3, inherently lacking kinase activity, becomes a potent mediator of cellular signaling upon dimerization with other HER family receptors, especially HER2. This interaction is pivotal in cells with amplified HER2, as it triggers the PI3K/AKT and MAPK pathways, crucial for cancer cell survival and proliferation (41, 78–80).

**Figure 3:**
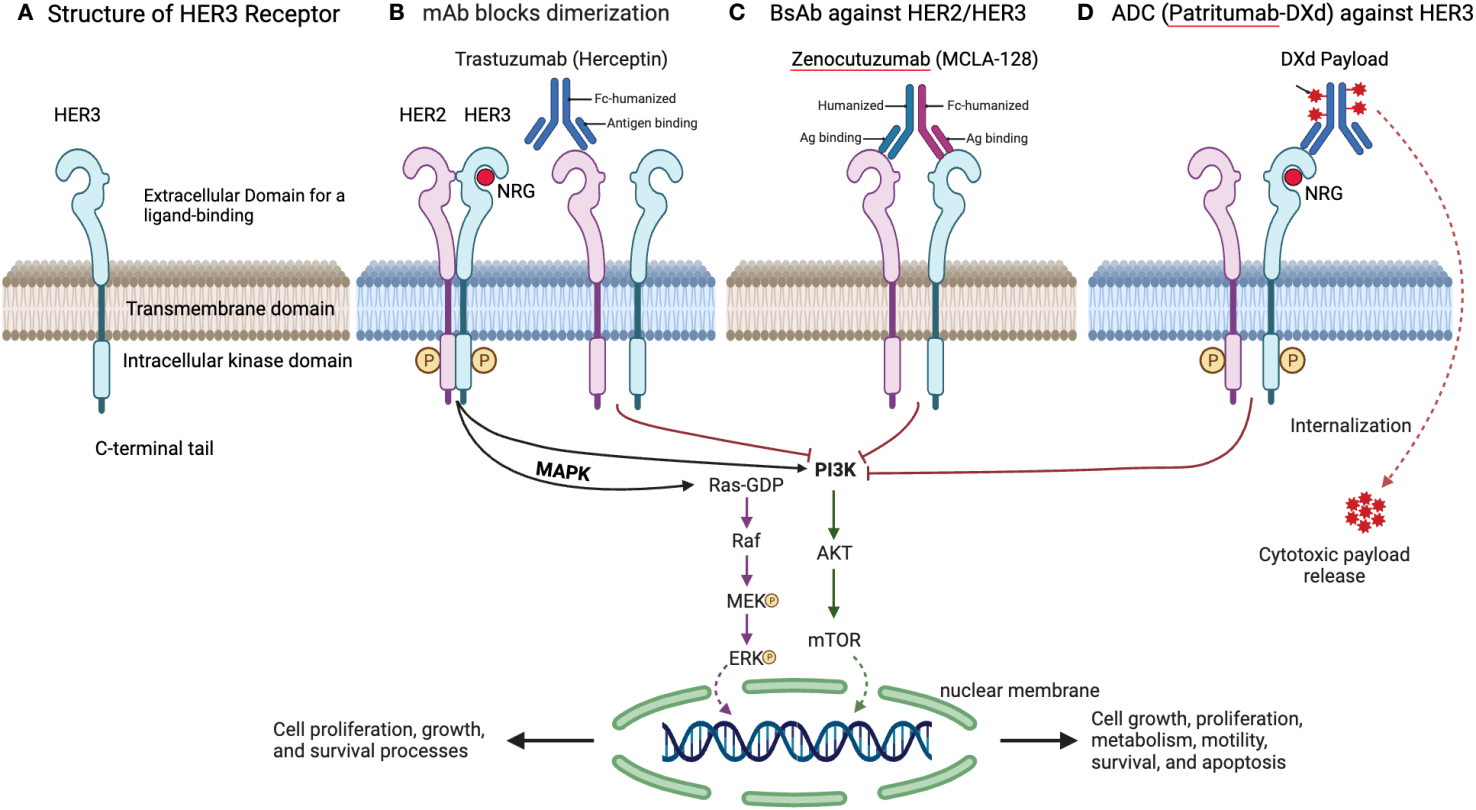
Overview of HER3 structure and targeting strategies. **(A)** Structure of HER3 Receptor. This detailed design of the HER3 receptor, including its extracellular domain for ligand binding, transmembrane domain, intracellular kinase domain, and C-terminal tail, is pivotal for its function and the development of targeted therapies. **(B)** mAb Blocks Dimerization. Trastuzumab, a mAb, inhibits HER2 dimerization, a necessary step for HER2/HER3 signaling, effectively blocking downstream oncogenic pathways. **(C)** BsAb against HER2/HER3. Zenocutuzumab (MCLA-128) demonstrates a dual-action approach by binding to both HER2 and HER3, potentially overcoming resistance arising from HER3 involvement. **(D)** ADC Targeting HER3. Patritumab-DXd’s targeted delivery of a cytotoxic payload to HER3-expressing cells offers a refined strategy for addressing tumors resistant to standard EGFR TKIs.

The strategic disruption of these signaling cascades is the cornerstone of targeted therapy against HER3. **Figure 3** illustrates several therapeutic approaches, including monoclonal antibodies (mAbs) like Trastuzumab, which inhibit HER2 dimerization; bispecific antibodies such as Zenocutuzumab (MCLA-128) that bind to both HER2 and HER3; and antibody-drug conjugates (ADCs) like Patritumab-DXd, which deliver cytotoxic drugs directly to HER3-positive cells, leveraging the receptor’s internalization to induce cell death (81).

Seribantumab, a monoclonal antibody that targets HRG-mediated activation of HER3, is under extensive investigation. Studies by Sequist et al. and Denlinger et al. have shown its potential in NSCLC and other solid tumors, indicating their role in overcoming resistance and improving patient outcomes (82, 83). The phase II study of MCLA-128, a full-length IgG1 bispecific antibody targeting HER2 and HER3, in patients with solid tumors (eNRGy) trial and early access program (EAP) are further evaluating Zenocutuzumab for its efficacy in NRG1 fusion-positive tumors, with pharmacokinetic analyses affirming the appropriateness of flat dosing for various solid tumors. Recent data from the eNRGy study and EAP have shown promising results in patients with NRG1 fusion-positive solid tumors, including NSCLC, pancreas cancer, breast cancer, and cholangiocarcinoma. The investigator-assessed objective response rate (ORR) was 34%, with responses observed in various tumor types. Additionally, the duration of response (DOR) was reported to be 9.1 months, indicating a robust and durable efficacy of Zenocutuzumab. Notably, the safety profile of Zenocutuzumab was well-tolerated, with grade ≥ 3 adverse events reported in less than 5% of patients. These findings suggest that Zenocutuzumab holds promise as a treatment option for advanced NRG1 fusion-positive cancers, offering potential benefits across different tumor histology. The ongoing phase II eNRGy trial and early access program continue to investigate Zenocutuzumab’s effectiveness in this patient population, providing hope for improved therapeutic options in the future (44, 84, 85).

GSK2849330, a pioneering monoclonal antibody that binds to HER3, preventing activation and subsequent downstream signaling, has shown a promising safety profile and efficacy in preliminary studies, including those with NRG1 expression, marking it as a potential new therapy for HER3-dependent tumors (86–88).

Patritumab (U3-1287), a fully human monoclonal antibody against HER3, competes with NRG for HER3 binding, hindering the proliferation and survival of tumor cells. Clinical trials have demonstrated its efficacy, particularly in NSCLC, with potential as a predictive biomarker for patient response to treatment (89, 90). Further research has validated its pharmacokinetics and safety in combination with other treatments, highlighting its significant role in the targeted cancer therapy (91–93).

Complementing these targeted therapies, ADCs like Patritumab-DXd have shown promising efficacy in phase II clinical trials, notably in NSCLC patients with resistance to EGFR inhibitors, including those with CNS metastases (94, 95). In an update on April 14, 2023, the ongoing phase III trial, HERTHENA-Lung02 (NCT05338970), suggested that Patritumab-DXd may be effective against various EGFR TKI resistance mechanisms, providing a new option for drug-resistant cancers. The study comprises approximately 560 patients with EGFR-activating mutations (exon 19 deletion or L858R) who progressed after 1 or 2 lines of EGFR TKI treatment, including a third generation TKI. Patients are randomly assigned to HER3-DXd or PBC treatment, with the primary endpoint being progression-free survival and the key secondary endpoint being overall survival. This trial has the potential to expand treatment choices for EGFR-mutated NSCLC patients confronting TKI resistance (https://doi.org/10.1158/1538-7445.AM2023-CT066).

Innovative combination strategies, such as pairing Patritumab-DXd with immune checkpoint inhibitors, are being explored to potentially enhance the overall anti-tumor effect, while also combining EGFR TKIs with HER3 inhibitors, as in the case of Osimertinib with Patritumab and Erlotinibotinib with Lumretuzumab, to achieve effective therapeutic outcomes and delay resistance (96, 97).

In conclusion, the dynamic targeting of HER3 with monoclonal antibodies, bispecific antibodies, and ADCs, as depicted in **Figure 3**, symbolizes a groundbreaking chapter in cancer treatment. Zenocutuzumab and Patritumab-DXd are at the vanguard of expanding treatment options, with ongoing trials confirming their potential to significantly enhance outcomes for patients facing HER3-mediated resistance in NSCLC.

## 5 Advances in HER3 targeting: from bench to bedside

The increasing acknowledgment of HER3’s role in oncogenesis has spurred intensive research into its therapeutic potential, spanning foundational preclinical studies to advanced clinical trials.

Preclinical studies highlight HER3’s significant impact on cell growth when overexpressed in various cancers (98). Activation by ligands such as HRG initiates vital cell survival pathways (42, 99). Given HER3’s limited kinase activity, its relationship with counterparts like HER2 becomes even more critical. The focus during the preclinical phase has been on devising strategies and developing compounds for HER3. The utilization of gene-editing tools like siRNA (38) and CRISPR (100), together with innovative HER3 inhibitors (44, 77, 84), suggests promising future therapies. Therapies targeting HER3, especially when paired with EGFR or HER2 treatments, demonstrate increased efficacy against resilient tumors (41).

*In vitro* studies offer detailed insights into HER3’s cellular behavior, with a notable correlation between higher HER3 expression and increased cell growth. Advanced molecular techniques have further underlined HER3’s central role in cellular signaling, particularly when interacting with HER2 (101). Techniques such as surface plasmon resonance have been instrumental in understanding HER3’s role in cancer progression.

Insights from animal models, especially xenografts, underscore the efficacy of HER3-targeted treatments. Observations indicate that inhibiting HER3 can lead to reduced tumor growth and extended survival rates (38, 75, 102). Studies have also associated heightened HER3 expression with aggressive tumor traits, including a propensity for metastasis and increased angiogenesis (37, 41).

In the realm of clinical trials, various HER3-targeting agents, including monoclonal antibodies like patritumab (81, 94, 95) and seribantumab (82, 103) have shown promising results. However, these agents are just a portion of the broader landscape. An extensive exploration of other antibodies under clinical evaluation can provide insights into their development, mechanisms, and therapeutic potential. Given the challenge of drug resistance, strategies combining EGFR TKIs with HER3 inhibitors are gaining traction (23, 31, 104, 105).

Precision medicine’s rise emphasizes treatments tailored to individual patient biomarkers, pushing for more personalized and potent NSCLC therapies (106). For a comprehensive overview of efforts and advancements in HER3-targeted treatments for NSCLC, refer to **Table 2**, which lists relevant clinical trials.

**Table 2:** Clinical trials involving HER3-targeted agents in NSCLC.

Study Title	Interventions	Phase	Study Design	Cases Enrolled	NCT Number	Primary Completion
A Study of MM-121 in Combination with Chemotherapy Versus Chemotherapy Alone in Heregulin Positive NSCLC	Drug: MM-121, Drug: Docetaxel	Phase 2	Allocation: Randomized; Intervention Model: Single Group. Masking: None. Primary Purpose: Treatment	153	NCT02387216	January 2, 2019
A Study of DB-1310 in Advanced/Metastatic Solid Tumors	Drug: DB-1310	Phase 1, Phase 2	Allocation: Non-Randomized; Intervention Model: Sequential. Masking: None. Primary Purpose: Treatment	287	NCT05785741	August 31, 2026
Herthena-Lung02: A Study of Patritumab Deruxtecan Versus Platinum-based Chemotherapy in Metastatic or Locally Advanced EGFRm NSCLC After Failure of EGFR TKI Therapy	Drug: Patritumab Deruxtecan, Drug: Platinum-based chemotherapy	Phase 3	Allocation: Randomized; Intervention Model: Parallel. Masking: None. Primary Purpose: Treatment	560	NCT05338970	August 4, 2026

## 6 Overcoming obstacles in HER3 targeting

HER3 offers significant therapeutic potential in oncology, yet its exploitation is riddled with challenges. The structural similarities within the ErbB receptor family, which includes EGFR (HER1), HER2, HER4, and HER3, lead to specificity issues. Drugs targeting HER3 might inadvertently influence other receptors, posing risks of reduced efficacy and unexpected complications. While monoclonal antibodies offer heightened specificity, there remains a concern about cross-binding within the ErbB family (107). Furthermore, even kinase inhibitors tailored for HER3 could impact other proteins, given the unique nature of HER3’s kinase domain. This highlights the importance of proteomic arrays and biomarker evaluations (42, 108).

Another hurdle is the adaptability of tumors, especially their propensity to develop secondary resistance. Such challenges are evident in anaplastic lymphoma kinase (ALK)-rearranged lung cancers, where mutations, alternative activation pathways, and epigenetic shifts are observed (109–112). Consequently, HER3-targeted treatments might encounter similar resistance mechanisms. It’s imperative to employ advanced genomic tools for continuous patient monitoring and to develop therapies that can preemptively counter resistance (113).

While combination therapies enhance potency, they may also escalate toxicity (114). Unexpected synergistic impacts or alterations in drug pharmacokinetics can lead to complications. Striking a balance between treatment efficacy and patient safety is crucial, necessitating regular health evaluations, adjusted dosing, comprehensive patient education, and robust supportive care.

In conclusion, effective HER3 targeting requires a comprehensive, patient-centric strategy to harness its therapeutic benefits without compromising safety.

## 7 Evolving horizons in HER3 targeting

The progress in HER3 targeting highlights the critical role of multidisciplinary collaboration. By amalgamating the insights of researchers, clinicians, and pharmaceutical companies, transformative strides in cancer care become possible. The emphasis is on crafting next-generation HER3 agents, optimizing specificity and efficacy.

One promising approach is coupling HER3 inhibitors with other treatment modalities, notably immunotherapies. These synergistic combinations enhance the immune system’s ability to recognize and eliminate cancer cells. In the context of NSCLC, the surge in HER3 inhibitor studies not only illuminates their therapeutic potential but also aids in pinpointing ideal patient candidates. These discoveries hold promise for reshaping treatment protocols for NSCLC and beyond.

As we reflect on the strides made, the horizon for HER3-focused treatments radiates with hope. This era is defined by groundbreaking innovations, holistic treatment blueprints, and an unwavering commitment to research, signifying a transformative phase in cancer care.

## 8 Conclusion

The advent of EGFR TKIs has been a game-changer for NSCLC treatments. However, resistance remains a significant challenge, particularly due to the upregulation of HER3. Combining HER3 targeting with EGFR TKIs emerges as a potent countermeasure against such resistance.

As the repertoire of HER3-targeted agents grows and their integration with therapies like immunotherapies becomes more refined, optimism for the future intensifies. Realizing the potential in this domain requires a multi-pronged approach: rigorous research, precise patient selection via advanced biomarkers, tactical treatment combinations, and a deepened comprehension of resistance pathways. With ongoing improvements in clinical trials and the development of predictive biomarkers, the potential of HER3 targeting in NSCLC shines brightly, fostering renewed optimism for surmounting EGFR TKI resistance.

## Author contributions

QC: Conceptualization, Data curation, Investigation, Project administration, Resources, Visualization, Writing – original draft, Writing – review & editing, Methodology. XZ: Data curation, Investigation, Methodology, Writing – original draft, Funding acquisition. GJ: Data curation, Investigation, Methodology, Writing – original draft, Formal analysis, Visualization. WM: Data curation, Formal analysis, Investigation, Visualization, Writing – original draft, Conceptualization, Project administration, Resources, Software, Supervision, Validation, Writing – review & editing.
