# Corrigendum: Targeting HER3 to overcome RGFR TKI resistance in NSCLC

**DOI:** 10.3389/fimmu.2024.1376045

**Published:** 2024-01-31

**Authors:** Qiuqiang Chen, Gang Jia, Xilin Zhang, Wenxue Ma

**Affiliations:** ^1^ Key Laboratory for Translational Medicine, The First Affiliated Hospital, Huzhou University, Huzhou, Zhejiang, China; ^2^ Department of Medical Oncology, Henan Provincial People’s Hospital, People’s Hospital of Zhengzhou University, Zhengzhou, Henan, China; ^3^ Department of Medicine, Moores Cancer Center, and Sanford Stem Cell Institute, University of California San Diego, La Jolla, CA, United States

**Keywords:** non-small cell lung cancer (NSCLC), epidermal growth factor receptor (EGFR), tyrosine kinase inhibitors (TKIs), receptor tyrosine kinases (RTKs), resistance, human EGFR3 (HER3), antibody-drug conjugates (ADCs), Patritumab Deruxtecan (HER3-DXd)

In the published article, there was an error in affiliation 1. Instead of “1”, it should be “3” only for the corresponding author Wenxue Ma. The correct affiliations of the authors are listed below.

Qiuqiang Chen^1*^, Gang Jia^2^, Xilin Zhang^1^, Wenxue Ma^3*^

^1^ Key Laboratory for Translational Medicine, The First Affiliated Hospital, Huzhou University, Huzhou, Zhejiang, China

^2^ Department of Medical Oncology, Henan Provincial People’s Hospital, People’s Hospital of Zhengzhou University, Zhengzhou, Henan, China

^3^ Department of Medicine, Moores Cancer Center, and Sanford Stem Cell Institute, University of California San Diego, La Jolla, CA, United States

The authors apologize for this error and state that this does not change the scientific conclusions of the article in any way. The original article has been updated.

